# 以额部肿物为首发症状的多骨累及的弥漫大B细胞淋巴瘤1例

**DOI:** 10.3760/cma.j.cn121090-20231225-00339

**Published:** 2024-06

**Authors:** 秀丽 郭, 敏 白, 彦凤 郗

**Affiliations:** 1 山西省肿瘤医院，中国医学科学院肿瘤医院山西医院，山西医科大学附属肿瘤医院病理科，太原 030013 Department of Pathology, Shanxi Province Cancer Hospital/Shanxi Hospital Affiliated to Cancer Hospital, Chinese Academy of Medical Sciences/Cancer Hospital Affiliated to Shanxi Medical University, Taiyuan 030013, China; 2 山西省肿瘤医院，中国医学科学院肿瘤医院山西医院，山西医科大学附属肿瘤医院血液科，太原 030013 Department of Hematology, Shanxi Province Cancer Hospital/Shanxi Hospital Affiliated to Cancer Hospital, Chinese Academy of Medical Sciences/Cancer Hospital Affiliated to Shanxi Medical University, Taiyuan 030013, China

患者，男，56岁，2021年8月因右额部皮下肿物15 d就诊。头部CT示右侧额骨骨质破坏，伴邻近皮下软组织肿胀，考虑恶性。额部肿物大小约5 cm×4 cm，表面皮肤光滑，肤色正常，无破溃，质略硬。肿物细针穿刺，苏木精-伊红（HE）染色结果显示大量深染异型的细胞，为正常淋巴细胞3～4倍及以上，细胞大，核质比高，染色质粗颗粒状，细胞恶性特征明显。细胞蜡块切片显示坏死背景中见大量异型细胞，为正常淋巴细胞的3倍以上，核圆形、卵圆形，部分呈分叶状，泡状核，染色质粗，部分细胞有2～4个靠近核膜的小核仁（图1A）。免疫细胞化学结果：CD20（弥漫+，图1B）、Ki-67（约70％+，图1C），AE1/AE3、CD3、Syn、CgA、CD56、TTF1、HMB45、Melan-A、SOX-10、P40阴性表达。右额部皮下肿物穿刺：恶性肿瘤细胞，考虑弥漫大B细胞淋巴瘤。患者由神经外科转入血液科。随后进行了彩超引导下右额部肿物穿刺活检：镜下见挤压的细胞结构欠清晰，因为有细胞学结果作为参考，因此免疫组化更有方向性。免疫组化结果：CD20（弥漫+ 图1D）、MUM1（约90％+）、Bcl-6（约80％+）、Ki-67（约95％+，图1E）、c-Myc（约10％+）、Bcl-2（约90％+）、P53（野生型+）、CD22（约95％+），CD3、CD10、CD5、CD30、CD19、Syn、AE1/AE3阴性表达。诊断：弥漫大B细胞淋巴瘤，非生发中心（Non-GCB）型。与细针穿刺结果一致。分子检测：MYC、BCL2、BCL6均未检测到断裂。骨髓活检：骨髓增生活跃，粒红比例大致正常，粒红二系均以偏成熟阶段细胞为主，巨核细胞数量及形态大致正常，分叶核为主。骨髓涂片：淋巴细胞占10％，未见异常淋巴细胞。骨髓免疫流式细胞术检测未见骨髓浸润。PET-CT：右侧额骨皮下代谢增高软组织肿块影，相邻骨质受侵犯；右侧眼眶外侧壁代谢增高软组织肿块，相邻上颌窦前壁及颧弓骨受侵；左侧颧突、双侧上颌骨、枕骨基底部、C5、T6、L1椎体多发骨代谢增高，部分骨质呈溶骨样改变。结果符合淋巴瘤征象，Deauville评分：5分。诊断为弥漫大B细胞淋巴瘤Ⅳa期，给予一线RCTOP（利妥昔单抗+环磷酰胺+吡柔比星+长春新碱+泼尼松）方案化疗2个周期。化疗后PET-CT较前：右侧额骨皮下软组织影消失，相邻颅骨呈虫蚀样改变，无代谢，其余病灶代谢均消失，Deauville评分：2分。全身PET-CT检查疗效评估为完全缓解。LDH水平从治疗前的653 U/L降至150 U/L（正常值120～250 U/L）。截至2023年10月，该患者已确诊弥漫大B细胞淋巴瘤2年余，共化疗9次，且每年的影像学检查都未发现活动性疾病或复发的迹象。

**图1:**
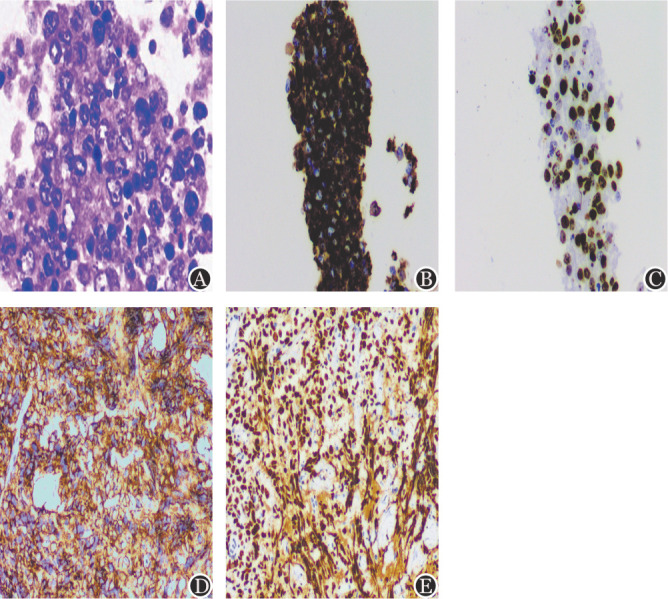
弥漫大B细胞淋巴瘤患者细胞蜡块及经超声引导下穿刺活检组织染色结果 **A** 肿瘤细胞体积为正常淋巴细胞的3倍以上，胞质少，嗜伊红，核圆形、卵圆形，部分呈分叶状，泡状核，染色质细，有2～4个靠近核膜的小核仁（HE，×400）；**B** CD20肿瘤细胞弥漫阳性（EnVision法，×200）；**C** Ki-67增殖指数约70％（EnVision法，×200）；**D** 活检组织免疫组化CD20肿瘤细胞弥漫阳性（EnVision法，×100）；**E** 活检组织免疫组化Ki-67增殖指数约95％（EnVision法，×100）

讨论：PET-CT显示该患者全身多发颅骨及椎骨病灶，右侧额部皮下软组织肿块，右侧眼眶外侧壁代谢增高软组织肿块，相邻上颌窦前壁及颧弓骨受侵。由于脊柱或上颌窦恶性淋巴瘤明确骨原发还是软组织原发存在困难，判断存在一定主观性，因此本例无法确定。本例骨淋巴瘤因发生部位罕见，且形成皮下肿物，主要应与以下疾病相鉴别：①皮肤转移性癌结节：男性以肺癌、胃癌多见，女性以肺癌、乳腺癌、卵巢癌多见，另转移性恶性黑色素瘤也较为常见。这类病例多有明确的病史，容易诊断。②原发性皮肤淋巴瘤：皮肤是仅次于胃肠道的结外淋巴瘤第二好发器官，表现为多发红色或紫红色丘疹、结节、肿块样皮损，与本例临床表现有较大差别。③原发性中枢神经系统淋巴瘤是指仅累及脑实质、脊髓、眼、颅神经及脑膜，无中枢神经以外部位受累的一类结外非霍奇金淋巴瘤，90％以上为弥漫大B细胞淋巴瘤。该患者PET-CT显示多发骨病变及软组织肿块，所以不考虑原发性中枢系统淋巴瘤。患者以额部皮下肿物就诊，未见皮肤损害，首先考虑转移性癌及恶性黑色素瘤可能性大，淋巴系统标志只作为排除性标志，免疫细胞化学结果考虑弥漫大B细胞淋巴瘤。超声引导下核芯针穿刺活检明确诊断弥漫大B细胞淋巴瘤，Non-GCB型。

